# *NOTCH-1* Gene Mutations Influence Survival in Acute Myeloid Leukemia Patients 

**DOI:** 10.31557/APJCP.2020.21.7.1987

**Published:** 2020-07

**Authors:** Salah Aref, Rasha Rizk, Mohamed El Agdar, Wafaa Fakhry, Maha El Zafrany, Mohamed Sabry

**Affiliations:** 1 *Hematology Unit, Clinical Pathology Department, Mansoura University Faculty of Medicine, Mansoura, Egypt. *; 2 *Medical Oncology Unit, Mansoura University Oncology Center, Mansoura University, Egypt. *

**Keywords:** * NOTCH-1*, mutations, AML, overall survival

## Abstract

**Background:**

Although *NOTCH-1 *gene mutations were reported to contributes to leukemogenesis in lymphocytic leukemias, its role in acute myeloid leukemia (AML) remains unclear. Therefor; this study was designed to determine the prevalence and clinical impact of* NOTCH-1* mutations in AML patients.

**Materials and Methods:**

In the current study, *NOTCH-1 *gene mutations were identified in Bone Marrow samples obtained from fifty primary AML patients before start of therapy using Sanger sequencing.

**Results:**

*NOTCH-1 *gene mutations were detected in 6 out of 50 AML cases (12%). The three mutations were (two mutations C7318A in the Pest domain exon 34); (another 2 in the Pest domain Del 7,344, ins *C7349*, *G7356A* and the last ones in the HD-N exon-26 (Del A4609). The clinical findings in the mutant *AML* (mu AML) patients did not significantly different as compared to the un mutated (unmut) AML patients. There is significant association between *CD7* aberrant expression and *NOTCH-1* mutations. The complete remission was significantly higher in unmut AML cases as compared to mut AML ones (P=0.024). Multivariate (Age; Gender; Bone Marrow Blast cells; *NOTCH-1* mutations) Cox regression analysis revealed that *NOTCH-1* mutation is an independent risk factor for AML overall survival (P<0.001). The OS in unmut AML group (21.2 months) was significantly longer as compared to mut AML one (1.2 months) (P<0.001).

**Conclusion:**

Our data indicate that *NOTCH-1 *gene mutations were detected in 12% of AML patients. These mutations displayed bad clinical outcome on AML patients. Therapeutic targeting of NOTCH-1 could be a potentially effective approach to combat master oncogenic drivers in AML.

## Introduction

Acute myeloid leukemia (AML) is a heterogeneous disorder characterized by impaired differentiation and increased proliferation of myeloid progenitors (blasts) in the bone marrow and peripheral blood. Acute myeloid leukemia (AML) is the most common acute leukemia in adults, accounting for ~80 percent of cases in this group. Recent studies have revealed that the disorder arises from a series of recurrent hematopoietic stem cell genetic alterations accumulated with age (Sun et al., 2016). 

Genetic alterations of genes in the NOTCH pathway have been noted in human malignancies both in hematopoietic and solid tumors. Most genetic alterations of NOTCH receptors in malignancies are observed in the *NOTCH-1* gene (Mao , 2016). *NOTCH-1* is a highly conserved and fundamental signaling system that mediates cell–cell interactions during animal development through highly context-dependent and cell-type-dependent effects on cell growth, fate determination and survival. Aberrations in *NOTCH-1* signaling or components of the signaling system underlie various human diseases including carcinogenesis ((Mao , 2016; Zheng et al., 2016). 

*NOTCH-1* encodes a transmembrane receptor which act as a ligand activated transcription factor. *NOTCH-1* signaling initiates when the ligand, from either the Jagged or Delta families, binds to the receptor and induces successive proteolytic cleavages, resulting in the release and nuclear translocation of the *NOTCH-1* intra-cellular domain (NICD). Inside the nucleus, the NICD assembles a transcriptional complex which interacts with the transcription factor CBF1/RBP-Jk, leading to de-repression/activation of CBF1- dependent target genes.18–20 Several members of the hairy/ enhancer of split (Hes) family of the basic helix–loop–helix proteins including HES1 are direct *NOTCH1*/CBF1 transcriptional targets. Besides this canonical pathway, *NOTCH1* also activates Deltex1 (DTX1), which binds to the ankyrin repeats of the NICD, likely antagonizing CBF1 binding (Arruga et al., 2014).

The *NOTCH-1* pathway is involved in chronic lymphocytic development (Aref et al., 2020) development and recurrent activating mutations in *NOTCH-1* contribute to T-lymphoblastic leukemias (Francis et al., 2017). *NOTCH-1* could be more expressed and activated in bone marrow, and such activation could be critical to mediate AML chemoresistance. *NOTCH-1* mutation leading to drug resistance and associated with relapsed/refractory AML (Takam Kamga et al., 2016). Other studies have described the involvement of *NOTCH-1* in microenvironment-mediated chemoresistance (Pisklakova et al., 2016). These results suggest that NOTCH signaling inhibition, by overcoming the stromal-mediated promotion of chemoresistance, may represent a potential therapeutic target for AML (Takam Kamga et al., 2016). In spite of that *NOTCH-1* signaling is actively involved in the regulation of myeloid development, its role in the myeloid leukemogenesis is less clear due to conflicting reports.

The aim of the current study was designed to determine the prevalence and clinical impact of *NOTCH-1* mutations among a cohort of AML Egyptian patients 

## Materials and Methods

*Patients and methods*

*Patients*

The current study was carried out on 50 patients with acute myeloid leukemia recruitment at Mansoura university oncology center in the period between 2014 - 2017. The age of the patients ranged from 24 to 59 years (24 females and 26 males). The AML diagnosis was based on the morphological, immunophenotyping and cytogenic basis. 

Ethical committee approval and informed consents were obtained from all included subjects. Adult patients with AML, were treated with 3 + 7 protocol consists of 3 days doxorubicin (45mg/m2) and 7 days cytarabine (100 - 200 mg/m^2^ IV continuous infusion over 24 hours). The mean follows up time of the studied AML patients was 24 months. The Clinical and laboratory data were recorded for every one case.

*Definition of complete remission (CR)*

CR in AML has been defined using the following criteria developed by an International Working Group

- Normal values for absolute neutrophil count (>1,000/μL) and platelet count (>100,000/μL), and independence from red cell transfusion.

- A bone marrow biopsy that reveals no clusters or collections of blast cells. 

- Extramedullary leukemia (eg, central nervous system or soft tissue involvement) must be absent.

*Inclusion criteria*

Patients with newly diagnosed primary AML 

Adult AML (age more than 18 years and less than 60 years).

*Exclusion criteria*

Promyelocytic leukemia (M3)

*Secondary AML*

*Methods*

All patients were subjected to full history taking, thorough clinical examination, and abdominal ultrasonography, routine laboratory Investigations (complete blood count, liver function tests, creatinine, lactic dehydrogenase, bone marrow aspiration, cytochemical stains, cytogenetic analysis and immunophenotyping) and specific investigation which includes *NOTCH-1* gene mutations detection by sanger sequencing method using ABI sequencer.

*NOTCH-1 gene mutations analysis*

The DNA was extracted using TRIzol agent (Invitrogen, Carlsbad, CA) from Fresh bone marrow samples. Then DNA amplification was done using PCR. The amplified target includes heterodimerization (HD) domain (exon 26), exon 34 (TAD domain) and exon 34 (pest domain) of *NOTCH-1* gene. The DNA amplification was done using the following primer pairs: Exon 26 FW: 5-GGAAGGCGGCCTGAGCGTGTC-3; exon 26 RV: 5-ATTGACCGTGGGCGCCGGGTC-3; exon 34 FW1: 5-CTGGCCTTTGAGACTGGC-3; exon 34 RV1: 5-GCTGAGCTCACGCCAAGGT-3; exon 34 FW2: 5-CAGATGCAGCAGCAGAACCTG-3; and exon 34 RV2: 5-AAAGGAAGCCGGGGTCTCGT-3. Cycling conditions were 35 cycles with annealing temperature 67.5º for exon 26, 63º for exon 34 TAD and 64º for exon 34 PEST domain.

*NOTCH-1 gene mutations detection*

The resultant PCR products were purified on Qiagen columns (Qiagen, Inc., Valencia, CA) and sequenced by *NOTCH-1* primers on ABI Prism 3700 DNA Analyzer using BigDye Terminator v3.1 Cycle Sequencing Kit (Applied Biosystems, Foster City, CA).

*Statistical Analysis*

The statistical analysis of data was done using excel program (Microsoft Office 2013) and SPSS (statistical package for social science) program (SPSS, Inc, Chicago, IL) version 20. Qualitative data were presented as frequency and percentage. Chi square and Fishers exact tests were used to compare groups. Quantitative data were presented by mean, SD or median and range. Comparisons between two groups were done using t-test or Man Whitney (for non-parametric). Kaplan-Meier curve was used to assess the overall survival and the log-rank test was used to compare the survival between the groups. To determine hazard ratios (HR) the Cox proportional hazard model (Cox-regression) was used. P-value less than 0.05 was considered statistically significant.

## Results

*Basic clinical data of AML patients’ group*

AML cases showed different clinical presentations at diagnosis; the most common was bleeding manifestations (76%), followed by fatigue (64%), pallor (56%), fever/infection (48%), weight loss (40%), lymphadenopathy (28%), splenomegaly (20%), hepatomegaly (16%) and the least presentation was CNS infiltration (12%). AML cases were classified according to FAB classification; 20% were M2, 56% were M4, 20% were M5, 4% were M6 and M3 cases was excluded (Table 1).

*NOTCH-1 Gene mutations status detection in studied AML patients’ group*

**Figure 1:**
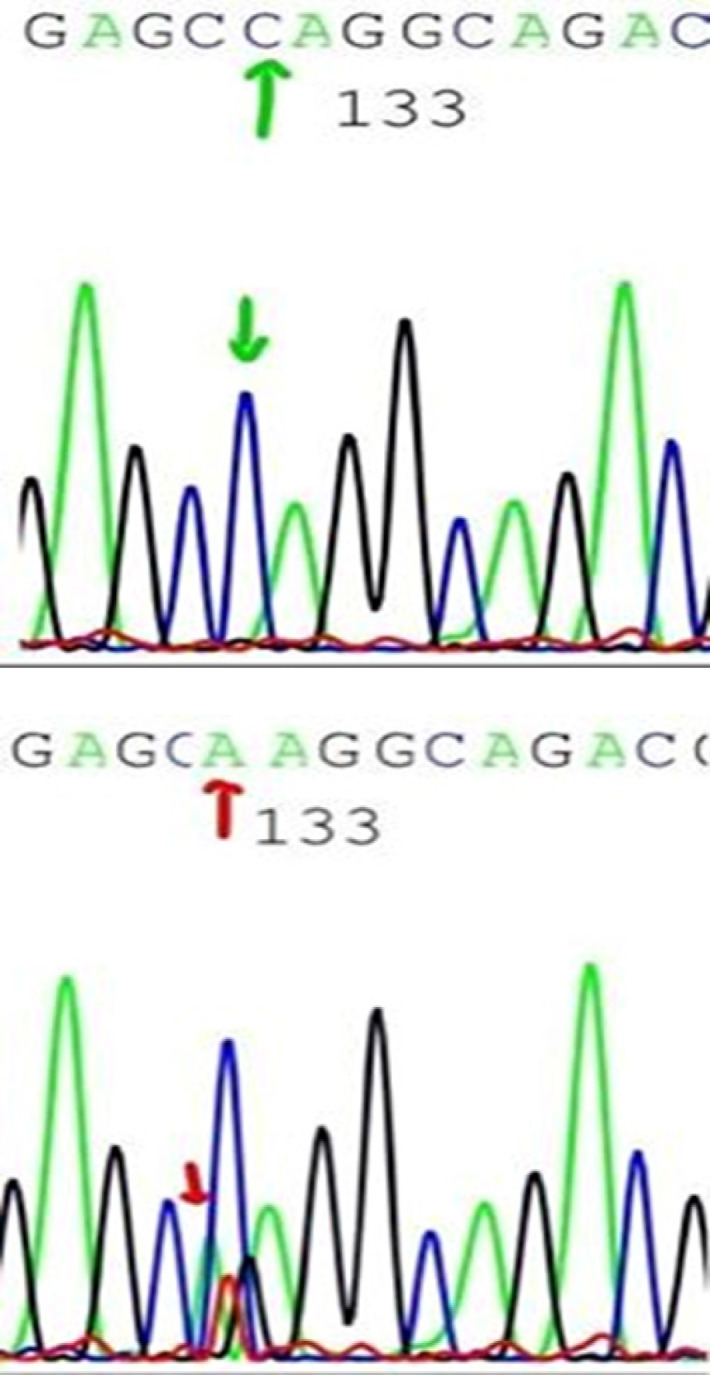
Showed Sequencing Results for Pest Domain, exon 34 in Case Number One

AML patients display *NOTCH-1* mutations in 6 cases out of 50 (12%). Two mutant cases were detected in the heterodimerization (HD) domain (exon 26) and 4 mutant cases were found in the proline, glutamic acid, serine, threonine-rich (PEST) domain (exon 34) of the *NOTCH-1* receptor which were all predicted to result in enhanced *NOTCH-1* signaling. Nucleotide and amino acid changes are shown in Table (2); Figure 1.

*Impact of NOTCH-1 Gene mutations on AML laboratory findings*

No significant differences were found in blood counts data and FAB subtypes between wild and mutant *NOTCH-1* groups in all studied AML groups. *NOTCH-1* mutated AML cases were associated with adverse cytogenetic risk, with significant association between cytogenetic risk and *NOTCH-1* mutations in studied AML patients (Table 3).

*Relation between NOTCH-1 Gene mutations and response to therapy*

Thirty-four cases of studied AML cases achieved CR (68%), 16 cases failed to achieve CR (32%), 10 were refractory (20%) and 6 died during induction therapy (12%). Those who achieved CR, 12 cases continued CR (70.6%) and 5 cases relapsed (29.4%). AML patients with *NOTCH-1* mutations failed to achieve CR, with significant difference from those with wild *NOTCH-1*. Mutant *NOTCH-1* cases were significantly associated with death in aplasia. All studied mutant *NOTCH-1* cases died during the study period, whereas, more than two third of wild *NOTCH-1* cases still alive by the end of the study, with statistically significant difference. 

*Analysis for overall survival of AML patients*

**Figure 2:**
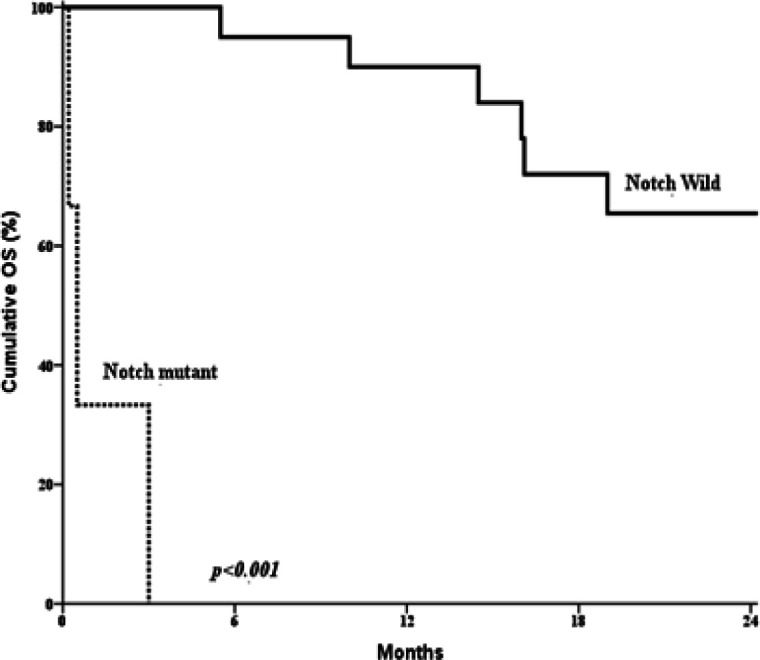
Kaplan-Meier Survival Curve Displaying a Significant Difference between AML Patients with Mutated *NOTCH-1 *Gene (n = 6) and Those with Unmutated ones (n = 44) (p = 0.001).

 During the entire period of the study (24 months), 18 cases died (36%), while 32 were alive (64%). All mutated *NOTCH-1* cases died during the study period, their mean overall survival (OS) was 2.6 months. Whereas, the mean OS for AML patients with wild *NOTCH-1* cases was 21.2 months. Mutant *NOTCH-1* cases showed significantly shorter OS when compared to AML patients with wild type (P<0.001). All AML patients with mutant *NOTCH-1* were refractory or died during induction therapy, therefore no DFS was calculated for them (Tables 4, 5 and Figure 2).

Cox proportional hazard of the clinical and laboratory data on the AML patient’s outcome

Cox regression analysis was conducted for prediction of OS within studied AML cases, using age, gender, clinical, laboratory, FAB subtypes, immunophenotyping and Notch mutation as covariates. The significant parameters in univariable analysis were introduce in multivariable. *NOTCH-1* mutations were considered as poor prognostic factor for prediction of shorter OS within studied AML cases (Table 6).

**Table 1:** Clinical and Laboratory Data in Wild and Mutated *NOTCH-1* Gene AML Patients

	AML patients with wild NOTCH-1 (n=44) No (%)	AML patients with Mutated NOTCH-1 (n=6) No (%)	*P* value
Splenomegaly	6 (13.6)	4 (66.7)	0.091
Hepatomegaly	6 (13.6)	2 (33.3)	0.422
Lymphadenopathy	14 (31.8)	0 (0)	0.534
CNS infiltration	4 (9.1)	2 (33.3)	0.33
Positive HCV antibodies	6 (13.6)	4 (66.7)	0.091
FAB			
M2	10 (22.7)	0 (0)	0.285
M4	26 (59.1)	2 (33.3)	
M5	6 (13.6)	4 (66.7)	
M6	2 (4.5)	0 (0)	
Aberrant CD7	1 (4.5)	2 (66.7)	0.18

Categorical data are expressed as number, percentage; compared using Fisher exact test.

**Table 2:** *NOTCH-1 *Gene Mutations Variants and Distributions in the Studied AML Cases

Detected 6 mutations	Domain	Nucleotide change	Amino acid change
Two mutations	Pest domain, exon 34	C7318A	Q2441k
One mutation		Del 7344, insC7349, G7356A	S2450-2451A
One mutations		C7550T	P2518L
One mutations	HD-N exon 26	G5011A	V1672I
One mutation		Del A4609	C1537L k1538Q

**Table 3:** Association between Cytogenetic Findings and *NOTCH-1* Gene Mutations in the Studied AML Patients

	Cytogenetic findings		Mutated *NOTCH-1*
	type	No	No
I	Recurrent cytogenic abnormalities [t (8, 21), Inv16, t(16, 16)]	20	0
II	Normal cytogenetic	20	0
III	Adverse cytogenetic [(-5,-7) , 11qr, Complex karyotyping]	10	6

**Table 4:** Clinical Outcome in Mutated versus Unmutated AML Patients

	Wild *NOTCH-1*	Mutated *NOTCH-1*	*P* value
	(n=44)	(n=6)	
	n (%)	n (%)	
Complete remission	34 (77.3)	0 (0)	0.029
Refractory disease	8 (18.2)	2 (33.3)	
Induction death	2 (4.5)	4 (66.7)	
Complete remission	34 (77.3)	0 (0)	0.024
Failure of CR	10 (22.7)	6 (100)	
Alive	32 (72.7)	0 (0)	0.037
Dead	12 (27.3)	6 (100)	

CR, Complete remission; Categorical data are expressed as number, percentage; compared using Fisher exact test

**Table 5:** Impact of* NOTCH-1* Gene Mutations on AML Overall Survival

	NOTCH-1 unmutated AML patients (N=44)	NOTCH-1 mutated AML patients (N=6)	*P* value
Two-years Cumulative OS* (%)	65.50%	0%	<0.001
Months (95 % CI**)	21.2 (18.6-23.9)	1.2 (0.2-2.9)	

*OS, overall survival; **CI, confidence interval; *P* value, <0.001(AML with wild *NOTCH-1 *vs those with mutated *NOTCH-1*)

**Table 6:** Cox Proportional Hazard Model for Prediction of OS within Studied AML Cases

Parameters	*P* value	OR	95% CI	
Age <40years vs ≥40 years	0.2	1.032	1.083	0.983
Gender Male vs Female	0.661	1.343	5.012	0.36
Total leucocytic count (X10^9^/L) ≥50,000/cmm Vs<50,000 /cmm	0.609	0.998	1.007	0.988
Hemoglobin (g/dL) <10 g/dl Vs≥10g/dl	0.554	1.129	1.687	0.756
Platelet count (X10^9^/L) ≥50,000/cmm Vs<50,000/cmm	0.391	1.001	1.004	0.998
Peripheral blasts (%) Positive Vs Negative	0.294	0.986	1.012	0.961
BM Blast (%) ≥50% Vs <50%	0.348	0.989	1.012	0.966
Infection Positive vs Negative	0.668	1.337	5.031	0.355
Bleeding manifestation Positive vs Negative	0.71	1.348	6.52	0.279
Splenomegaly Positive vs Negative	0.60	1.524	7.359	0.316
Hepatomegaly Positive vs Negative	0.315	2.26	11.091	0.46
Lymphadenopathy Positive vs Negative	0.269	0.31	2.478	0.039
CNS infiltration Positive vs Negative	0.221	2.679	12.975	0.553
FAB (M0, M1, M2, M4, M5)	0.182	1.587	3.126	0.806
NOTCH1 mutations Wild vs Mutated AML	0.001	5.287	22.265	1.028

OR, odds ratio; CI, confidence interval

## Discussion

In the current study *NOTCH-1* gene mutations were detected in 6 out of 50 (12%) AML patients. The previous reports demonstrated that the *NOTCH-1* mutations in AML patients ranged from zero up to 8.3% (Tohda, 2014; Kim et al., 2009). This prevalence was different from those reported in T-cell acute lymphoblastic leukemia and chronic lymphocytic leukemia, in which more than half the cases displayed *NOTCH-1* mutations (Aref et al., 2020a; Aref et al., 2020b; Tohda, 2014). 

All patients with *NOTCH-1* mutations were detected in M4 and M5 AML FAB subtypes. This finding was in consistent with that reported by previous researchers who detected *NOTCH-1* mutation in different FAB subtypes (M1, M3, M4 and M5a) (Kannan et al., 2013). 

In addition, *NOTCH-1* mutation was detected in cell line derived from an AML M5 patient at relapse and M4 cell line (Takam Kamga et al., 2019). *NOTCH-1* mutations have also been described in patients with chronic myelomonocytic leukemia (CMML) (Klinakis et al., 2011). The recombinant *NOTCH-1* ligand proteins could alter AML blast cells into macrophage-like cells morphologically (Yatim et al., 2012). However, other studies supported that *NOTCH-1* mutations were associated with AML without morphological maturation (M0 or M1) (Takam Kamga et al., 2019; Noronha et al., 2019; Kannan et al., 2013; Sliwa et al., 2014; Lutherborrow et al., 2014). This difference was attributed to rare prevalence in *NOTCH-1* mutations in AML, and the fact that *NOTCH-1* signaling inhibited a monocytic/granulocytic differentiation program in an early multipotent progenitor (Takam Kamga et al., 2019; Lobry et al., 2014).

Further examination of AML patients in the present study revealed enlargement of the spleen in 4 out of 6 mutant *NOTCH-1* patients. This may be due to extramedullary hematopoiesis as they were FAB M4 and M5. 

Bone marrow blasts in our mutant *NOTCH-1* AML patients showed wide variation, it ranged from 40 to 90% (Kannan et al., 2013). Similar finding was reported by previous studies which stated that bone marrow blasts in *NOTCH-1* mutant AML patients was ranged between 68 and 97% (Kannan et al., 2013).

Four out of 6 AML cases in the present study showed aberrant expression of *CD7*. This was in consistent with previous reports which was reported that *CD7* was consistently highly expressed in all mutant *NOTCH-1* AML specimens (Takam Kamga et al., 2019). Others showed significant correlation between *NOTCH-1* hyperexpression and aberrant expression of *CD7* (Sliwa et al., 2014). *CD7*, is known to be expressed by a considerable proportion of immature AMLs (Estey, 2012). 

The previously reported mutations occur mostly in the heterodimerization (HD) domain (exon 26, 27) and proline, glutamic acid, serine, threonine-rich (PEST) domain (exon 34) of the *NOTCH 1* receptor (Noronha et al., 2019; Hales et al., 2014), which were all predicted to result in enhanced *NOTCH-1* signaling (Steinbuck and Winandy, 2018; Malecki et al., 2006). Four out of 6 cases with *NOTCH-1* mutant AML cases in the present study were detected in PEST domain and the other 2 cases were detected in HD domain. This agreed with previous studies (Tohda, 2014; Noronha et al., 2019; Shiba et al., 2010). 

Five AML cases displayed *NOTCH-1* gene mutations in the current study have adverse cytogenetic data. This finding claimed that *NOTCH-1* mutations might be a bad prognostic marker in AML cases. However, others reported that *NOTCH-1* expression may be a relevant prognostic marker in intermediate risk AML (Xu et al., 2011). The association of *NOTCH-1* mutations with adverse cytogenetic risk may be due to common dysregulation that affect DNA repair and stem-cell maintenance or certain aberrations in the stroma-microenvironment which might influence the evolution of malignant conditions and exhibit distinct genotypic and phenotypic features.

In the present study AML patients have *NOTCH-1* mutations express failure of CR. This may be due to conventional chemotherapies, often become ineffective due to the heterogeneity of leukemia cells (Desai, 2015). While others showed similar outcome associated with *NOTCH-1* mutation in adult AML (Zhou, 2012). On the other hand, Pediatric studies, showed an excellent outcome in *NOTCH-1* mutated AML patients (Gao et al., 2014; Yeh et al., 2016). 

The difference in outcome association with *NOTCH-1* could be due to small number of mutant *NOTCH-1* patients in each study, association of comorbidities, different race and different treatment protocols.

In our study, mutant *NOTCH-1* cases showed significantly shorter OS when compared to those with wild *NOTCH-1* cases. As all mutated *NOTCH-1* cases were refractory or died during induction therapy, no DFS was calculated for them. Cox regression analysis was conducted for prediction of OS within studied AML cases, using age, gender, BM blasts, *NOTCH-1* mutation as covariates. *NOTCH-1* mutations were considered as poor prognostic factor for prediction of shorter OS within studied AML cases. Association between inferior outcome and *NOTCH-1* mutations or hyperexpression was also noticed by many authors in human samples (Kannan et al., 2013; Sliwa et al., 2014; Lutherborrow et al., 2014; Lobry et al., 2018; Czemerska et al., 2015; Kamga et al., 2018). This might be explained on the basis that *NOTCH-1* mutations lead to truncation of the C-terminal PEST domain which result in decreased degradation of intracellular portion of *NOTCH-1* receptor and consequently higher *NOTCH-1* signaling (De Falco et al., 2018). 

Based on our findings we conclude that the frequency of *NOTCH-1 *gene mutations among AML patients was 12% and that AML patients harbored *NOTCH-1 *gene mutation displayed bad clinical outcome. Wide scale studies are recommended in order to validate the results detected in the present study.

## References

[B1] Aref S, El-Agdar M, Salama O (2020a). Clinical value of NOTCH1 mutations detection among chronic lymphocytic leukemia patients. Asian Pac J Cancer Prev.

[B2] Aref S, El Agdar M, Salama O (2020b). Significance of NOTCH1 mutations détections in T-acute lymphoblastic leukemia Patients. Cancer Biomarkers.

[B3] Arruga F, Gizdic B, Serra S (2014). Functional impact of NOTCH1 mutations in chronic lymphocytic leukemia. Leuk Lymphoma.

[B4] Czemerska M, Pluta A, Szmigielska-Kaplon A (2015). Jagged-1: a new promising factor associated with favorable prognosis in patients with acute myeloid leukemia. Leuk Lymphoma.

[B5] De Falco F, Del Papa B, Baldoni S (2018). IL-4-dependent Jagged1 expression/processing is associated with survival of chronic lymphocytic leukemia cells but not with Notch activation. Cell Death Dis.

[B6] Desai UN (2015). Enhancement of the cytotoxic effects of Cytarabine in synergism with Hesperidine and Silibinin in Acute Myeloid Leukemia: An in-vitro approach. J Cancer Res Ther.

[B7] Estey EH (2013). Acute myeloid leukemia: 2013 update on risk-stratification and management. Am J Hematol.

[B8] Francis OL, Chaudhry KK, Lamprecht T (2017). Impact of Notch disruption on myeloid development. Blood Cancer J.

[B9] Gao C, Liu SG, Zhang RD (2014). NOTCH1 mutations are associated with favourable long-term prognosis in paediatric T-cell acute lymphoblastic leukaemia: a retrospective study of patients treated on BCH-2003 and CCLG-2008 protocol in China. Br J Haematol.

[B10] Hales EC, Taub JW, Matherly LH (2014). New insights into Notch1 regulation of the PI3K-AKT-mTOR1 signaling axis: targeted therapy of γ-secretase inhibitor resistant T-cell acute lymphoblastic leukemia. Cell Signal.

[B11] Kamga P, Resci F, Dal Collo G (2018). Prognostic impact of notch signaling in acute myeloid leukemia (AML). Blood.

[B12] Kannan S, Sutphin RM, Hall MG (2013). Notch activation inhibits AML growth and survival:a potential therapeutic approach. J Exp Med.

[B13] Kim YR, Eom HS, Min CK (2009). Analysis of NOTCH1 extracellular juxtamembrane expansion mutations in acute leukemias and multiple myelomas. APMIS.

[B14] Klinakis A, Lobry C, Abdel-Wahab O (2011). A novel tumor-suppressor function for the Notch pathway in myeloid leukemia. Nature.

[B15] Lobry C, Oh P, Mansour MR (2014). Notch signaling: switching an oncogene to a tumor suppressor. Blood.

[B16] Lutherborrow M, Bryant A, Jayaswal V (2014). Expression profiling of cytogenetically normal acute myeloid leukemia identifies MicroRNAs that target genes involved in monocytic differentiation. Am J Hematol.

[B17] Maillard I, Pear WS (2018). Can genetics resolve what Notch does in HSCs?. Blood.

[B18] Malecki MJ, Sanchez-Irizarry C, Mitchell JL (2006). Leukemia-associated mutations within the NOTCH1 heterodimerization domain fall into at least two distinct mechanistic classes. Mol Cell Biol.

[B19] Mao L (2016). NOTCH Mutations: Multiple faces in human malignancies. Cancer Prev Res.

[B20] Noronha EP, Marques LVC, Andrade FG (2019). T-lymphoid/myeloid mixed phenotype acute leukemia and early T-cell precursor lymphoblastic leukemia similarities with NOTCH1 mutation as a good prognostic factor. Cancer Manag Res.

[B21] Pisklakova A, Grigson E, Ozerova M (2016). Anti-myeloma effect of pharmacological inhibition of Notch/gamma-secretase with RO4929097 is mediated by modulation of tumor microenvironment. Cancer Biol Ther.

[B22] Shiba N, Kanazawa T, Park MJ (2010). NOTCH1 mutation in a female with myeloid/NK cell precursor acute leukemia. Pediatric Blood Cancer.

[B23] Sliwa T, Awsa S, Vesely M (2014). Hyperexpression of NOTCH-1 is found in immature acute myeloid leukemia. Int J Clin Exp Pathol.

[B24] Steinbuck MP, Winandy S (2018). A review of notch processing with new insights into ligand-independent notch signaling in T-Cells. Front Immunol.

[B25] Sun Y, Shen H, Xu T (2016). Persistent DNMT3A mutation burden in DNMT3A mutated adult cytogenetically normal AML patients in long- term remission. Leuk Res.

[B26] Takam Kamga P, Dal Collo G, Resci F (2019). Notch signaling molecules as prognostic biomarkers for acute myeloid leukemia. Cancers (Basel).

[B27] Takam Kamga P, Dal Collo G, Resci F (2019). Notch signaling molecules as prognostic biomarkers for acute myeloid leukemia. Cancers (Basel).

[B28] Takam Kamga P, Bassi G, Cassaro A (2016). Notch signaling drives bone marrow stromal cell-mediated chemoresistance in acute myeloid leukemia. Oncotarget.

[B29] Tohda S 2014) NOTCH1 signaling roles in acute myeloid leukemia cell growth and interaction with other stemness-related signals. Anticancer Res.

[B30] Xu X, Zhao Y, Xu M (2011). Activation of notch signal pathway is associated with a poorer prognosis in acute myeloid leukemia. Med Oncol.

[B31] Yatim A, Benne C, Sobhian B (2012). NOTCH1 nuclear interactome reveals key regulators of its transcriptional activity and oncogenic function. Mol Cell.

[B32] Yeh CH, Bellon M, Pancewicz-Wojtkiewicz J (2016). Oncogenic mutations in the FBXW7 gene of adult T-cell leukemia patients. Proc Natl Acad Sci U S A.

[B33] Zhang J, Ye J, Ma D (2013). Cross-talk between leukemic and endothelial cells promotes angiogenesis by VEGF activation of the Notch/Dll4 pathway. Carcinogenesis.

[B34] Zheng B, Yu SF, Del Rosario G et al (2018). An anti-CLL-1 antibody-drug conjugate for the treatment of acute myeloid leukemia. Clin Cancer Res.

[B35] Zhou L (2012). Myeloproliferation and hematopoietic stem cell dysfunction due to defective Notch receptor modification by O-fucose glycans. Semin Immunopathol.

